# Single-position OLIF combined with percutaneous posterior fixation under O-arm navigation for the treatment of grade II lumbar spondylolisthesis: clinical and radiologic outcomes

**DOI:** 10.3389/fsurg.2025.1676460

**Published:** 2025-11-17

**Authors:** Zhi-da Chen, Song-song Wu, Yuan-jie Jiang, Hui Liu, Zhuan-zhi Huang, Tao-yi Cai, Bin Lin

**Affiliations:** 1 Department of Orthopaedics, The 909th Hospital, School of Medicine, Xiamen University, Zhangzhou, China; 2 Department of Orthopaedics, The Fifth Affiliated Hospital, Southern Medical University, Guangzhou, China

**Keywords:** spondylolisthesis, spinal fusion, surgical navigation systems, surgery, OLIF

## Abstract

**Introduction and aim:**

Grade II lumbar spondylolisthesis is frequently accompanied by segmental instability, intervertebral disc degeneration, and nerve root compression. When conservative management fails, surgery is generally warranted. Therefore, we conducted a retrospective study to evaluate the clinical and radiologic outcomes of single-position OLIF combined with percutaneous posterior fixation under O-arm navigation for the treatment of Grade II lumbar spondylolisthesis.

**Materials and methods:**

A retrospective analysis was conducted on 87 patients with Grade II lumbar spondylolisthesis who underwent single-position OLIF combined with percutaneous posterior fixation under O-arm navigation. The affected segments were as follows: 17 cases at L2, 26 cases at L3, and 44 cases at L4. Data were collected on operation duration, blood loss, hospital stay, radiological and clinical outcomes (VAS, ODI, SF-36, intervertebral disc height, slippage rate, lumbar lordosis angle, cross-sectional area, and sagittal diameter), Bridwell classification, and complications.

**Results:**

The mean operation duration was 118.7 ± 22.4 min, with an average blood loss of 83.6 ± 27.4 ml. All patients had regular follow up with an average duration of 29.5 ± 9.2 months. The VAS scores, ODI, and SF-36 at 3 months postoperatively and final follow-up showed significant improvement compared to preoperative scores (*P* < 0.05). The IDH, SR, and LLA were significantly improved at 3 days, 12 months, and at the final follow-up compared to preoperative values (All *P* < 0.01). Mean cross-sectional area improved significantly from 87.40 ± 29.59 mm^2^ preoperatively to 132.42 ± 33.53 mm^2^ at 12 months follow-up (*P* < 0.001). The mean sagittal diameter at 12 months follow-up 9.23 ± 2.87 mm showed statistically significant difference compared to preoperative measurements 5.25 ± 2.67 mm. 94.3% (82/87) of patients achieving Bridwell Grade I bone fusion. A total of 348 pedicle screws were implanted with an overall accuracy rate of approximately 98.9%. Complications were minimal, with 3 cases of psoas weakness that resolved to normal muscle strength within 2 weeks.

**Conclusions:**

It offers significant minimal invasiveness, accuracy in screw placement, and sustained reconstruction of lumbar sagittal plane, with low complication rates and high fusion success.

## 1 Introduction

Lumbar spondylolisthesis is a common degenerative disease with an incidence rate of 5.4%–11.5% (1, 2). Its primary clinical manifestations include chronic low back pain and intermittent claudication, which can result in disability in severe cases. Grade II lumbar spondylolisthesis frequently presents with nerve root compression and spinal instability, and thus often requires surgical intervention (3). Lumbar interbody fusion is the most common surgical treatment for lumbar spondylolisthesis (4). At present, posterior lumbar interbody fusion (PLIF) and transforaminal lumbar interbody fusion (TLIF) are widely adopted for lumbar spondylolisthesis. These procedures effectively restore vertebral alignment, relieve nerve compression, and maintain spinal stability (5). However, they also involve considerable surgical trauma, substantial paraspinal muscle damage, and a relatively high incidence of postoperative low back pain (6, 7). The extreme lateral interbody fusion (XLIF) procedure is approached via the retroperitoneal space and psoas muscle to access the lateral aspect of the spine, where the intervertebral disc is excised and replaced with a fusion implant. The preservation of the anterior and posterior longitudinal ligaments inherent to this technique allows for the placement of larger interbody implants than traditional posterior lumbar interbody fusion procedures. Consequently, this can restore disc height and facilitate indirect neural decompression (8). Nonetheless, the transpsoas approach inherent to XLIF carries risks, including potential damage to the psoas muscle and lumbosacral plexus. These complications may cause reduced lower extremity strength and localized sensory deficits (9). Additionally, the disease location near the iliac crest can obstruct the surgical view, presenting another limitation (10).

Oblique lateral lumbar interbody fusion (OLIF), first described by Mayer, traverses the space between the retroperitoneal psoas muscle and the abdominal aorta to reach the anterolateral margin of the vertebral body. This approach reduces injury to the psoas muscle and lumbosacral nerve roots while achieving decompression effectiveness similar to XLIF (11–13). However, conventional OLIF typically requires repositioning the patient from supine to prone during surgery for supplemental pedicle screw fixation. This repositioning necessitates re-draping and re-sterilization, thereby increasing both operative time and the risk of contamination. Recently, the O-arm and intraoperative navigation techniques have become increasingly important in spinal surgery (14). Compared with freehand techniques or traditional fluoroscopy, these new methods significantly enhance the accuracy of pedicle screw and cage placement, thereby reducing the risk of malposition (15). Recent studies have reported the feasibility of directly inserting pedicle screws in the lateral position without changing the posture (16). Consequently, in this study, we adopted single-position OLIF with O-arm navigation combined with posterior fixation to treat Grade II lumbar spondylolisthesis. This approach leverages both the accurate positioning enabled by O-arm navigation and the minimally invasive nature of OLIF, allowing anterior and posterior procedures to be performed in a single position. Therefore, we conducted a retrospective analysis of cases to evaluate the clinical and radiological outcomes of this technique.

## 2 Material and methods

### 2.1 Patient selection

All patients underwent lumbar spine x-rays, 3D-Computed Tomography (3D-CT), and magnetic resonance imaging (MRI) to determine the presence of lumbar spondylolisthesis. Patients included in the study had grade II lumbar spondylolisthesis, characterized by: (1) significant low back pain, radiating pain in the lower limbs, numbness and intermittent claudication. (2) failure of conventional conservative treatment for at least 3 months; and (3) single-segment spondylolisthesis at the L2-L4 vertebral level. Exclusion criteria included: (1) severe spinal canal stenosis or massive intervertebral disc herniation. (2) history of previous lumbar or abdominal surgeries. (3) severe systemic comorbidities contraindicating surgery. (4) less than 12 months of follow-up.

This retrospective case series was approved by the Ethics Committee of our institution, and all participants provided informed consent. Eighty-seven patients who met the following inclusion and exclusion criteria were enrolled in the study between January 2018 and December 2023. All patients received single-position OLIF combined with percutaneous posterior fixation under O-Arm navigation. Among them, 49 were male and 38 were female, with an average age of 67.9 ± 7.4 years (range, 46–78 years). The types of spondylolisthesis included 54 cases of isthmic spondylolisthesis and 33 cases of degenerative spondylolisthesis. The affected segments were as follows: 17 cases at L2, 26 cases at L3, and 44 cases at L4. All procedures were carried out by experienced spinal surgeons, and data collection spanned from initial surgery to final follow-up.

### 2.2 Operative procedure

All patients underwent combined approach surgery using general anaesthesia. Patients were positioned in the right lateral decubitus position. The lower limbs were flexed to reduce abdominal tension. A navigation reference arc was placed approximately 2 inches lateral to the iliac crest. The reference array was registered to enable real-time navigation based on the first CT scan (O-Arm, Medtronic). The entry point for the pedicle screws is identified along the line connecting the lateral border of the superior articular process and the midline of the transverse process. A hand awl is used to create the screw trajectory, after which a probe confirms the bony walls. Pedicle screws (Medtronic) were inserted bilaterally under O-arm fluoroscopic guidance.

**Figure 1:**
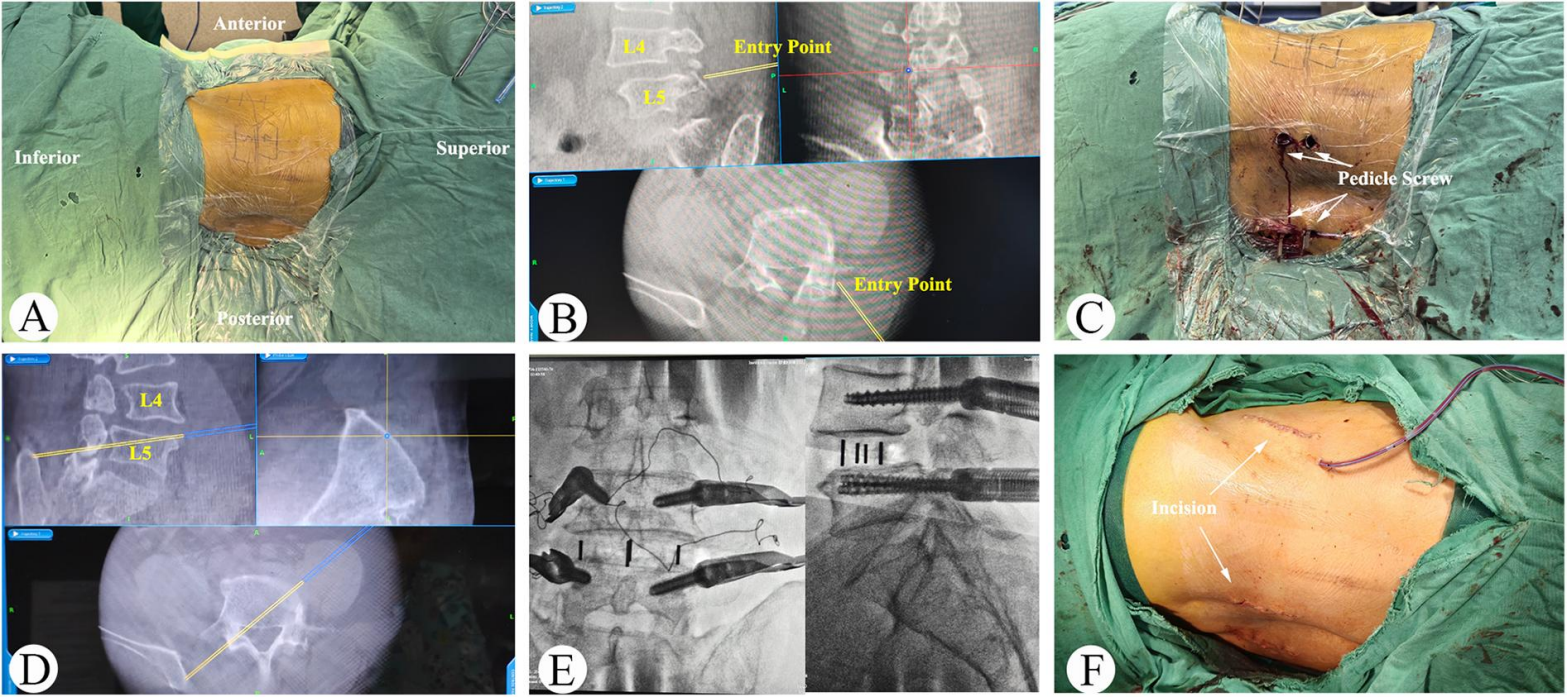
Single-position OLIF combined with percutaneous posterior fixation under O-arm navigation for the treatment of grade II lumbar spondylolisthesis. **(A)** The patient was positioned in right lateral decubitus position. **(B,C)** Percutaneous pedicle screws were inserted bilaterally under O-arm fluoroscopic guidance. **(D)** O-arm navigation was utilized for localization while accessing the intervertebral space. **(E)** OLIF cage was inserted under navigational assistance. **(F)** Surgical incision appearance.

An oblique incision was made 3 cm anterior to the midpoint of the target intervertebral disc space. Three muscular layers of the external oblique, the intra-abdominal oblique, and the transverse abdominis muscles were bluntly dissected and the retroperitoneal space was directly exposed by fingers of surgeon. Navigation was utilized for localization while accessing the intervertebral space in front of the psoas muscle. A probe was inserted down to the disc. The guidewire was then passed through the probe, followed by sequential dilation to displace the surrounding tissues. A retractor was placed over the dilators. The annulus fibrosis was incised, and the intervertebral disc nucleus along with the cartilaginous endplates were removed. Allogeneic bone was placed in the central cavity of cage and the implant was attached to the navigated inserter. The cage was inserted using an orthogonal maneuver under navigational assistance. x-ray was performed to confirm the cage's position and to acquire intraoperative images for percutaneous pedicle screw fixation. Two titanium rods of appropriate length were pre-bent and then installed. On one side, the distal pedicle screws were first tightened to secure the rod. Using a distractor along the rod's direction, the slipped vertebra was repositioned under compression or distraction. The proximal screws were then tightened. The same procedure was repeated on the contralateral side. x-ray confirms proper alignment. The surgical site was irrigated with saline, and the layers were closed sequentially. Operative procedure are shown in Figure 1.

### 2.3 Postoperative management and observation indicators

All patients received intravenous drip antibiotics, pain relief and prevention of deep vein thrombosis. And the patients were encouraged to ambulate with the assistance of a lumbosacral brace 3 days after operation. This was continued for an average of 8–10 weeks postoperatively.

### 2.4 Outcome measures

Intraoperative observations were recorded for blood loss and duration of operation. Patients received regular follow-ups every three months in the hospital's outpatient clinic. Clinical outcomes were evaluated using the Visual Analogue Scale (VAS), Oswestry Disability Index (ODI), the 36-Item Short-Form Health Survey (SF-36) and complications. Fusion outcomes were graded using the Bridwell classification system (17).

X-rays and CT scans were used to evaluate intervertebral disc height (IDH), slippage rate (SR), and lumbar lordosis angle (LLA). Measurement of the IDH using the modified Farfan method (18): H1 and H2 are the vertical distances from the anterior and posterior points of the lower endplate of the upper vertebral body to the superior endplate of the lower vertebral body, while h1 and h2 are the vertical distances from the anterior and posterior points of the upper endplate of the lower vertebral body to the inferior endplate of the upper vertebral body. IDH = (H1 + H2 + h1 + h2)/4. Measurement of SR using the Meyerding scale (19). The percentage is determined by measuring the length of the upper endplate of the lower vertebral body (a) and the distance between the point where this line intersects the extension of the posterior walls of the superior and inferior vertebral bodies (b), expressed as a/b. The LLA was formed by the angle between a line drawn parallel to the superior endplate of L1 and a line drawn parallel to the inferior endplate of S1. MRI scans were taken to assess changes in the cross-sectional area and sagittal diameter of the thecal sac. The accuracy of percutaneous screws was evaluated immediately after surgery using the Gertzbein-Robbins system (GRS) (20).

### 2.5 Statistical analysis

All statistical analyses were performed using SPSS 22.0 (SPSS Inc., Chicago, IL, USA). The Shapiro–Wilk test was used to assess whether the data conformed to a normal distribution. Metric data conforming to a normal distribution are presented as x ± s. Comparisons between preoperative and postoperative VAS, ODI, SF-36, IDH, SR, and LLA were made using the paired *t*-test. Repeated measures ANOVA was used to compare different time periods within the same group. A difference was considered statistically significant at *P* < 0.05.

## 3 Results

All patients successfully underwent the surgery. The average operation duration was 118.7 ± 22.4 min (range, 108–190 min). Intraoperative blood loss ranged from 56–158 ml, with a mean of (83.6 ± 27.4) ml. The average length of hospital stay was 4.3 ± 0.7 days. Patients were followed for a mean period of 29.5 ± 9.2 months. Detailed information of the patients was shown in Table 1.

**Table 1:** Detail information of the patients.

Variable	Value
No. of patients	87
Male [*n* (%)]	49 (56.3)
Female [*n* (%)]	38 (43.7)
Age [year (range)]	67.9 ± 7.4 (46–78)
Types of spondylolisthesis	
Isthmic spondylolisthesis [*n* (%)]	54 (62.1)
Degenerative spondylolisthesis [*n* (%)]	33 (37.9)
Affected segments	
L2/L3/L4 [*n*]	17/26/44
Operation duration [min (range)]	118.7 ± 22.4 (108–190)
Intraoperative blood loss [ml (range)]	83.6 ± 27.4 (50–150)
Hospital stay [d (range)]	4.3 ± 0.7 (3–5)
Follow-up [month (range)]	29.5 ± 9.2 (16–60)

### 3.1 Clinical efficacy and complications

The mean VAS score was 7.4 ± 2.7 preoperatively. It decreased to 2.8 ± 0.6 at one week, 1.8 ± 0.5 at 3 months, and 0.5 ± 0.2 at the last follow-up. Similarly, ODI scores showed a marked decline following the surgery. The preoperative ODI score was 65.8 ± 18.4, significantly improved to 30.5 ± 9.7 one week postoperatively (*P* < 0.001). The improvement was sustained and enhanced over time, with scores decreasing to 13.7 ± 8.6 at 3 months (*P* < 0.001 compared to preoperative and one-week scores) and further decreasing to 11.4 ± 5.2 at the final follow-up (*P* < 0.001 compared to all earlier measurements). Additionally, SF-36 scores improved from 73.5 ± 6.4 preoperatively to 112.7 ± 4.1 at the last follow-up (*P* < 0.001 compared to preoperative scores). These changes in VAS, ODI, and SF-36 scores were shown in Table 2.

**Table 2:** The clinical efficacy evaluation of patients pre and postoperation.

Items	Pre-op	1w Post-op	3-mon follow-up	Final follow-up	*F* value	*P* value
VAS	7.4 ± 2.7	2.8 ± 0.6	1.8 ± 0.5	0.5 ± 0.2	34.04	<0.001
ODI	65.8 ± 18.4	30.5 ± 9.7	13.7 ± 6.6	11.4 ± 5.2	37.57	<0.001
SF-36	73.5 ± 6.4	98.2 ± 6.9	105.9 ± 5.4	112.7 ± 4.1	67.16	<0.001

The surgical procedure demonstrated a low complication rate, with 3 cases (3.4%) presenting psoas weakness that resolved to normal muscle strength within 2 weeks. Furthermore, no surgical site infection, ureter injury, retrograde ejaculation, spinal nerve injury, major vessel injury, urinary tract injury, or hardware failure was observed during surgery.

### 3.2 Radiological evaluation

Tables 3, 4 summarize the radiological evaluation of patients pre- and postoperation. The IDH, SR, and LLA were significantly improved at 3 days, 12 months, and at the final follow-up compared to preoperative values (All *P* < 0.01). Preoperatively, the mean cross-sectional area was 87.40 ± 29.59 mm^2^, which was significantly increased to 135.73 ± 32.86 mm^2^ at 3 days postoperative (*P* < 0.001). At 12-months post-surgery, the cross-sectional area remained stable at 132.42 ± 33.53 mm^2^, with no statistically significant changes from the 3 days postoperative measurement (*P* > 0.05). The preoperative sagittal diameter was 5.25 ± 2.67 mm and the 3 days postoperative sagittal diameter was 9.44 ± 3.05 mm. There was a statistically significant difference between the preoperative and 3 days postoperative sagittal diameter. The 12-months postoperative sagittal diameter was 9.23 ± 2.87 mm, with statistically significant changes from the 3 days postoperative measurement (*P* > 0.05). A total of 348 pedicle screws were implanted in all 87 patients. The assessment of the screw placement accuracy showed: Grade A in 344 screws and Grade B in 4 screws, resulting in an overall accuracy rate of approximately 98.9%. Bone union was achieved between 3 and 6 months postoperatively, with an average time of 5.5 ± 1.4 months. At the final follow-up (beyond 12 months), 94.3% (82/87) of patients achieved grade I fusion, and 5.7% (5/87) achieved grade II fusion.

**Table 3:** The radiological evaluation of patients pre and postoperation.

Items	Pre-op	3d Post-op	12-mon follow-up	Final follow-up	*F* value	*P* value
IDH (mm)	6.05 ± 1.42	11.28 ± 0.61	11.43 ± 0.65	11.08 ± 0.68	62.46	<0.001
SR (%)	34.35 ± 6.87	4.05 ± 1.76	4.42 ± 2.18	4.38 ± 1.86	115.90	<0.001
LLA (°)	36.18 ± 2.88	48.28 ± 3.93	46.86 ± 1.76	46.16 ± 1.07	32.84	<0.001

**Table 4:** The cross-sectional area and sagittal diameter evaluation of patients pre and postoperation.

Items	Pre-op	3d Post-op	12-mon follow-up	*F* value	*P* value
Cross-sectional area (mm^2^)	87.40 ± 29.59	135.73 ± 32.86	132.42 ± 33.53	5.33	0.018
Sagittal diameter (mm)	5.25 ± 2.67	9.44 ± 3.05	9.23 ± 2.87	5.08	0.021

**Figure 2:**
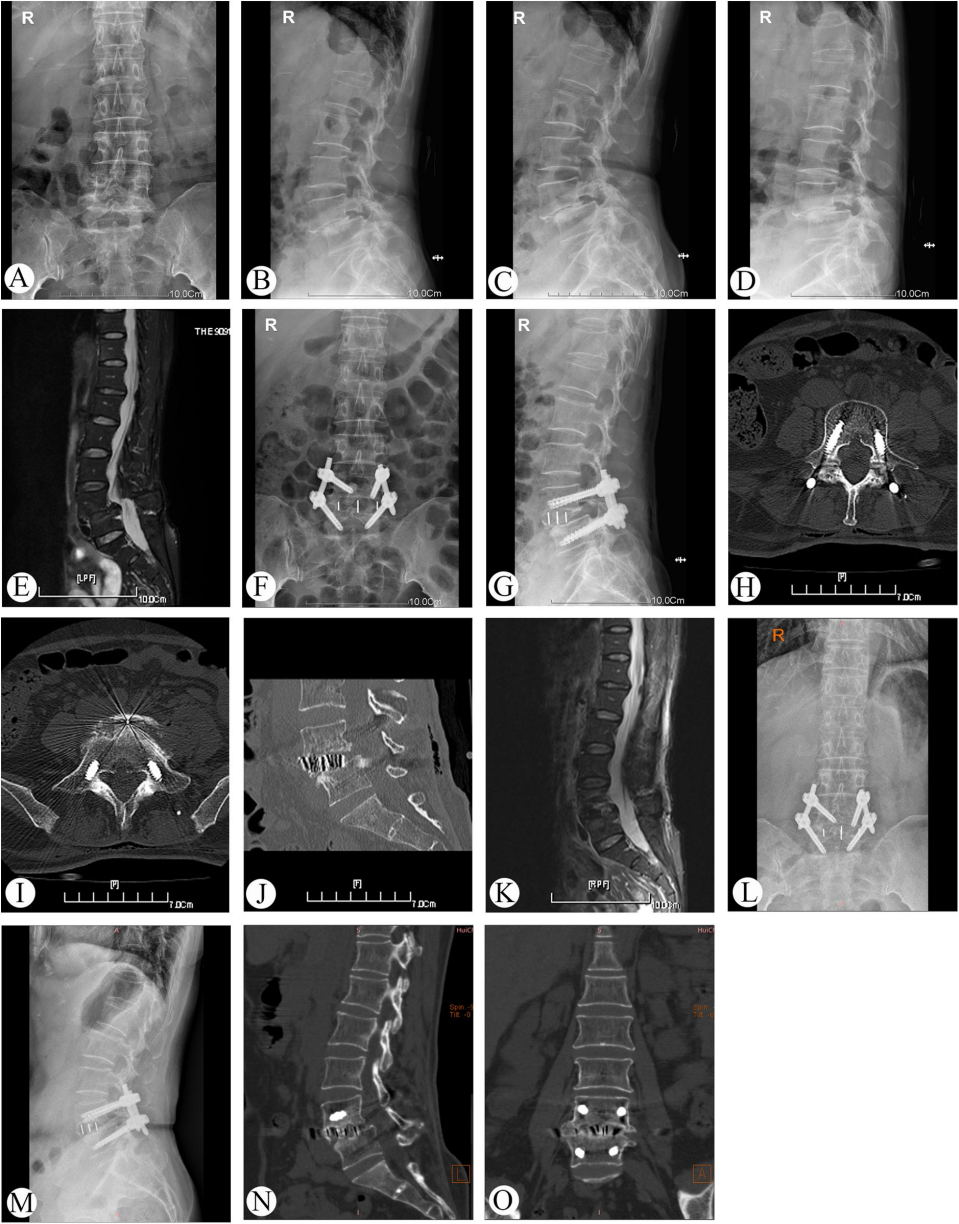
Preoperative, postoperative, and follow-up radiographs of a 56-year-old female patient who had grade II lumbar spondylolisthesis managed by single-position OLIF combined with percutaneous posterior fixation with O-arm navigation (case 13). **(A–E)** x-ray and MRI before operation. **(F–K)** x-ray, CT, and MRI at 3 days after operation. **(J–O)** x-ray and CT at 2 years follow-up.

Representative cases are shown in Figure 2 (Case 13).

## 4 Discussion

### 4.1 Management of grade II lumbar spondylolisthesis

Grade II lumbar spondylolisthesis is frequently accompanied by segmental instability, intervertebral disc degeneration, and nerve root compression. When conservative management fails, surgery is generally warranted (3). Surgical objectives are to reestablish spinal stability, decompress neural elements, and restore sagittal alignment. Conventional posterior PLIF/TLIF allows direct exposure of the spondylolisthetic segment, facilitates nerve root decompression, achieves satisfactory realignment of the slipped vertebra, and provides immediate spinal stability (18). However, these posterior techniques are relatively invasive, result in significant soft tissue injury to the paraspinal muscles, and are associated with a high incidence of postoperative low back pain.

Recently, minimally invasive lumbar interbody fusion techniques have been increasingly used to treat patients with lumbar spondylolisthesis (21). Oblique lateral lumbar interbody fusion (OLIF), an approach accesses the spine between the abdominal anterior vessel and the psoas muscle, is considered the solution to the limitations of ALIF, PLIF and XLIF (22, 23). OLIF is a minimally invasive surgical method for treating lumbar degenerative diseases, featuring advantages such as less bleeding, minor trauma, rapid patient recovery, short hospital stay, and a low incidence of infection (11). For patients with lumbar spondylolisthesis, however, supplemental posterior fixation affords greater segmental stability and reduces the incidence of cage subsidence, migration, and other procedure-related complications (24). And adequate anterior release combined with posterior distraction facilitates reliable reduction of the slipped segment and helps restore global spinal alignment. Currently, OLIF protocols for spondylolisthesis typically require two separate positions: lateral decubitus for the interbody fusion and prone for percutaneous pedicle-screw fixation. This intra-operative repositioning lengthens surgical time and may increase anesthesia-related risks. With recent advances in robotic assistance and navigation, pedicle-screw placement accuracy now exceeds 95% (25). Therefore, we employed a single-position OLIF with percutaneous pedicle-screw instrumentation under O-arm navigation for the management of grade II lumbar spondylolisthesis. This approach enables precise disc-space localization, clearly delineates safe working corridors within complex anatomy, and enhances overall surgical accuracy while eliminating the need for patient repositioning.

### 4.2 Advantages of single-position OLIF combined with percutaneous posterior fixation under O-arm navigation for grade II lumbar spondylolisthesis

Conventional OLIF combined with percutaneous posterior fixation requires intra-operative repositioning from the lateral decubitus to the prone position, which is both time-consuming and potentially associated with increased peri-operative risk (26). Some surgeons have adopted OLIF with percutaneous pedicle screw fixation performed in the lateral position (26, 27). However, pedicle screw insertion under lateral fluoroscopic guidance is technically challenging. Most spine surgeons are less familiar with pedicle screw insertion under lateral fluoroscopic guidance, and the oblique view can compromise spatial orientation. Intraoperative three-dimensional navigation has emerged as an effective strategy to counteract this limitation by providing real-time, anatomically accurate feedback. O-arm navigation achieved higher accuracy in percutaneous pedicle screw placement than conventional C-arm fluoroscopy (28). In addition, navigation guidance significantly decreased the number of fluoroscopic shots (29). In the present series, overall pedicle-screw accuracy reached 98.9%.

The selection of this approach includes the following: (1) Patients with lumbar grade Ⅰ and Ⅱ spondylolisthesis, especially with intervertebral stenosis and discogenic pain, but without severe spinal stenosis; (2) Sagittal imbalance requiring reconstruction of lumbar lordosis; (3) Elderly patients who cannot tolerate decompression fusion and internal fixation. The technique combines the advantages of navigation and OLIF, and has the following advantages: (1) Minimally invasive: OLIF was performed via the retroperitoneal approach and under the channel to avoid damaging the posterior paraspinal and ligament complex. The O-arm navigation system was used to achieve accurate positioning and screw placement. Anterior incision was only 3–4 cm, and the incision was only 5 small holes of about 1 cm, with minimal muscle damage and less bleeding. The incidence of postoperative iatrogenic low back pain was lower than posterior PLIF/TLIF surgery (7). Besides, the single position also avoided the risk of secondary draping and position change. In this group, the intraoperative blood loss was only 83.6 ± 27.4 ml, with VAS scores of 0.5 ± 0.2 at the final follow-up. Kim et al. reported that the blood loss was significantly less in single position OLIF group than in typical OLIF group (131.94 ± 95.40 vs. 270.00 ± 238.64, *P* = 0.003) (30). (2) Indirect decompression and slip reduction: OLIF can be placed in a larger interbody cage and directly spread the vertebral space. The significance of intervertebral height restoration lies in reducing the thickness of the posterior longitudinal ligament and the yellow ligament, achieving the expansion of the cross-sectional area, and increasing the height and area of the intervertebral foramen (31, 32). This indirect decompression mechanism can also promote partial natural reduction of the slipped vertebral body. In addition, O-arm navigation provides three-dimensional real-time imaging, which can dynamically adjust the position of the cage during the operation, ensuring that the cage is located the anterior 1/3 of the vertebral body. In our cases, the average increase in postoperative IDH was 5.03 mm. The SR average decreased by 29.97% and the LLA average increased by 9.98° at the final follow-up. Moreover, this surgical technique avoids the dura mater and nerve root before and after the operation, reducing the risk of nerve injury and cerebrospinal leakage. (3) High fusion rate: The cartilage endplates were fully processed through OLIF surgery, which can provide an excellent fusion environment (33). However, the incidence of cage-related complications in stand-alone OLIF is about 4.4%–8.7% (31, 34). Displacement and subsidence of cage will affect the fusion effect. This technique combined with posterior percutaneous fixation provides three-column stability, reducing the risk of cage subsidence or migration. All patients achieved bony fusion within 6 months postoperatively, and 94.3% achieved grade I fusion and 5.7% reached grade II fusion at finalfollow-up. (4) Sagittal plane reconstruction: Lumbar spondylolisthesis affect lumbar lordosis due to the decrease of the intervertebral height and local lordotic angle the responsible segment. Insufficient reconstruction of lumbar lordosis after operation will lead to adjacent segment degeneration, functional disorders, and residual pain (35, 36). Lumbar lordosis plays a significant role in maintaining sagittal balance, and loss of lordosis can cause sagittal imbalance, anterior displacement of the center of gravity, resulting in chronic low back pain (37). OLIF can increase lumbar lordosis by placing a wide, angled wedge cage, and correct the lordosis angle. Correction of the lordosis angle is also related to the position of the cage. The farther forward the cage is positioned, the more pronounced the correction of the anterior protrusion (38). Reduction and compression of combined posterior fixation leverages the lordosis angle further increase by using the cage as a fulcrum to make separated articular processes aggregated and relaxed anterior longitudinal ligament tensioned. LLA in our patients increased by an average of 9.98° at final follow-up, which effectively restored the physiological lumbar lordosis and maintained the sagittal balance of the lumbar spine. (5) Low complication rate: The incidence of complications related to OLIF surgery ranges from 0%–48.3% (31, 35). Common complications are manifested as endplate destruction, transient psoas muscle weakness, and numbness in the anterior medial thigh and inguinal area. In this cohort, only 3 cases had slight left psoas weakness, and muscle strength returned to baseline within 2 weeks.

### 4.3 Limitation

This study's retrospective nature, limited sample size, and lack of long-term follow-up highlight the need for further investigation. Future research should focus on prospective, multicenter studies with larger cohorts to validate these findings and enable direct comparisons with alternative surgical methods.

## 5 Conclusion

In conclusion, single-position OLIF combined with O-arm navigation and percutaneous pedicle screw fixation is a safe and effective technique for the treatment of Grade II lumbar spondylolisthesis. This technique presents several notable advantages, including minimal invasiveness and enhanced accuracy in screw placement, which contribute to high rates of lumbar slippage reduction and fusion. Additionally, it successfully restores intervertebral disc height and the cross-sectional area of the thecal sac, facilitating indirect decompression. This surgical approach is associated with significant improvements in both radiographic parameters and clinical outcomes, making it a valuable option for the management of Grade II lumbar spondylolisthesis.

## Data Availability

The raw data supporting the conclusions of this article will be made available by the authors, without undue reservation.
